# Dashboards to Improve Extractability of Cardiovascular Indicators in a Learning Health Care System: Mixed Methods Study

**DOI:** 10.2196/71978

**Published:** 2025-12-16

**Authors:** Anna G M Zondag, Karin R Jongsma, Wouter W van Solinge, Michiel L Bots, Robin W M Vernooij, Saskia Haitjema, UCC-CVRM Study Group, Gert J de Borst

**Affiliations:** 1 Central Diagnostic Laboratory, University Medical Center Utrecht, Utrecht University, Heidelberglaan 100, Utrecht, 3508 GA, The Netherlands, 31 887569376; 2 Julius Center for Health Sciences and Primary Care, University Medical Center Utrecht, Utrecht University, Utrecht, The Netherlands; 3 Department of Nephrology and Hypertension, University Medical Center Utrecht, Utrecht, The Netherlands; 4 See Acknowledgements

**Keywords:** cardiovascular risk management, cardiovascular disease, guideline adherence, clinical dashboards, feedback, learning health care system

## Abstract

**Background:**

Cardiovascular risk management (CVRM) guidelines have been developed to evaluate and manage all patients at higher cardiovascular risk, being either symptomatic or still asymptomatic. Although these guidelines have long existed, adherence varies. A learning health care system (LHS) could address adherence by continuously analyzing routine care data to inform and improve health care practice. Dashboards may be used to inform clinicians on the care provided and potentially improve structured registration of CVRM indicators in electronic health records (EHRs).

**Objective:**

We evaluated whether the implementation of dashboards in our LHS led to changes in the structured registration of cardiovascular indicators in patients at increased risk of cardiovascular disease (CVD).

**Methods:**

In our mixed methods study, patients who visited the University Medical Center Utrecht between January 2022 and November 2023, the period during which the dashboard was implemented, were included. We assessed the extractability of the CVRM indicators (ie, BMI, blood pressure, smoking status, medical CVD history, lipid levels, glycated hemoglobin, hemoglobin, and the estimated glomerular filtration rate), stratified by department. We compared the extractability of the indicators with the extractability before the Utrecht Cardiovascular Cohort-Cardiovascular Risk Management (UCC-CVRM) LHS was initialized and with the period during which the UCC-CVRM was protocolized, but without the use of dashboards. To explain our quantitative findings and to gain a deeper understanding of how the dashboards were viewed and perceived, we conducted semistructured interviews with clinicians and analyzed these thematically.

**Results:**

The extractability of CVRM indicators among 8941 first hospital visits remained low and stable during the period in which the dashboards were used. Overall, hemoglobin (5344/8941, 59.8%) and estimated glomerular filtration rate (5682/8941, 63.5%) were most often extractable, and patients’ CVD history (1946/8941, 21.4%) and smoking status (2543/8941, 28.4%) were the least extractable. Compared to the protocolized UCC-CVRM, indicators were up to 45% less extractable, meaning that CVRM indicators were less often registered in structured fields of the EHR. Interviews with clinicians (N=5) revealed that the low extractability could be attributed to unclear responsibility for CVRM, lack of harmonized agreements for registration in EHRs, perceived challenges related to the EHR system (eg, some structured fields were not easily accessible), time constraints, and habits (eg, maintaining habitual ways of working that are perceived to best suit their workflow).

**Conclusions:**

Dashboards did not improve the registration of CVRM indicators in structured fields of the EHR. This was explained by perceived organizational, technical, and operational issues, such as unclear responsibility for CVRM care, suboptimal technical knowledge and limitations of the EHR system, and time constraints. Our findings provide guidance on what aspects to consider for the extractability of CVRM indicators to be improved, which will be beneficial for both clinical practice and scientific research using real-world data.

## Introduction

Guidelines summarize and evaluate available evidence with the aim of assisting health care professionals in proposing the best management strategies for an individual patient with a given condition. Cardiovascular risk management (CVRM) guidelines have been developed to support health care professionals in the prevention of cardiovascular disease (CVD) based on individual patient characteristics [1]. These guidelines include cardiovascular risk indicators that should be assessed regularly in all symptomatic and asymptomatic patients at risk of CVD, such as smoking status, serum lipids, and physical measurements (eg, BMI), which also allow for an estimation of the individual 10-year absolute CVD risk, which may help for tailored interventions on an individual level. Yet, compliance with these guidelines varies between specialties [1].

Previous studies have shown that the systematic and uniform registration of CVRM indicators leads to improved guideline adherence and decreased the risk of missing patients with an indication for treatment [2]. However, manually scanning through the electronic health record (EHR) to identify eligible patients and the systematic and uniform registration of all indicators was noted to be laborious and time-consuming and, therefore, unsustainable [2]. Currently, digitalization of this process (ie, identification of eligible patients and automatic extraction of CVRM indicators from structured fields of the EHR) has made this less laborious, yet the quality of routine care data might hamper automatic extraction as these data are commonly unstructured and incomplete [3].

In the University Medical Center (UMC) Utrecht in the Netherlands, the Utrecht Cardiovascular Cohort-CardioVascular Risk Management (UCC-CVRM) was set up in 2014 as a learning health care system (LHS) to inform practice and improve structured and uniform registration of cardiovascular risk factors [4]. The setup of an LHS may lead to improvements in CVD care, as an LHS allows for the rapid translation of acquired insights (eg, compliance with clinical guidelines) into changes in clinical practice by providing feedback to clinicians. Since 2022, participating departments of the UCC-CVRM LHS regularly received clinical dashboards, including aggregated metrics on the presence of CVRM indicators in structured fields of the EHR.

However, it is unclear whether these dashboards improved structured registration of clinical indicators, and previous studies on the effect of dashboards are inconclusive [5-7]. Dashboards may induce behavior change by raising awareness (psychological capability), prompting reflection (motivation), and reducing barriers to action (opportunity). Currently, however, differing outcomes regarding behavior change in response to such feedback to clinicians are observed [8]. Previous systematic reviews collectively demonstrate that drawing firm conclusions about the effectiveness of dashboards in health care is challenging due to marked heterogeneity across studies [6,8]. This variability spans several dimensions: clinical settings (eg, primary care, mental health, and transplant care), patient populations, indicators used, target outcomes (eg, prescribing rates, treatment adherence, and diagnostic reporting), and the design and presentation of the dashboards themselves. Some dashboards were implemented as stand-alone tools, whereas others were part of broader multicomponent interventions. Visual formats also differed, ranging from simple traffic light coding to complex graphical displays [5,6]. Therefore, this study aimed to evaluate the extent to which the implementation of dashboards has led to changes in the structured registration of cardiovascular indicators in patients with an increased risk of CVD by quantitative analysis of the extractability of CVRM indicators from structured fields of the EHR and qualitative analysis of views and perceptions of clinicians on the dashboards.

## Methods

### Study Design

**Figure 1.**
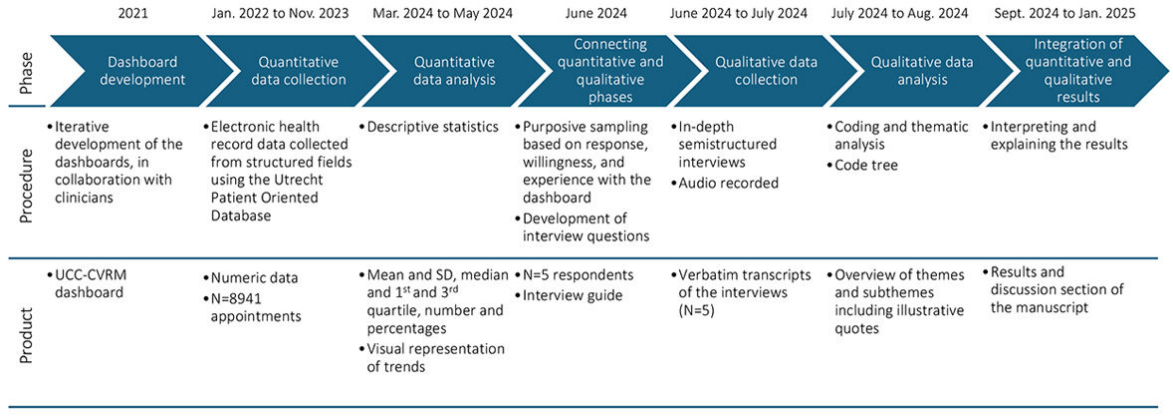
Diagram of the mixed methods design of this study. Adapted from a study by Ivankova et al [9]. UCC-CVRM: Utrecht Cardiovascular Cohort-Cardiovascular Risk Management.

This study was conducted using a sequential explanatory mixed methods study design (Figure 1). The reason we opted for this design was that this 2-phase design starts with a quantitative phase and is followed by a qualitative phase to explain and supplement the quantitative findings [9]. This approach fits best with what we intended to achieve in our study, namely, to first quantify the CVRM extractability, and then, based on these results, we aimed to explain our findings by interviewing clinicians, the end users of the dashboards.

### Dashboard Development and Design

We developed the UCC-CVRM dashboards using the data visualization software Power BI Desktop [10]. During the developmental phase of the dashboards, approximately 1 year before implementation, we had several meetings with clinicians from all participating departments to adjust the patient selection on which the data extraction for visualization in the dashboards would be based, if necessary, and to discuss the content of the dashboards. During this early phase, the initial goals and key functionalities of the dashboards were defined based on identified clinical needs and priorities. Following this internal development, a first set of prototype dashboards was presented to all participating departments. During these meetings, we invited clinicians from each department to critically assess the layout, content, and relevance of the selected indicators. Discussions focused on both the clinical usefulness of the dashboards and the patient selection. Feedback received during these sessions led to refinements in both the visual presentation and the underlying patient selection criteria.

**Figure 2.**
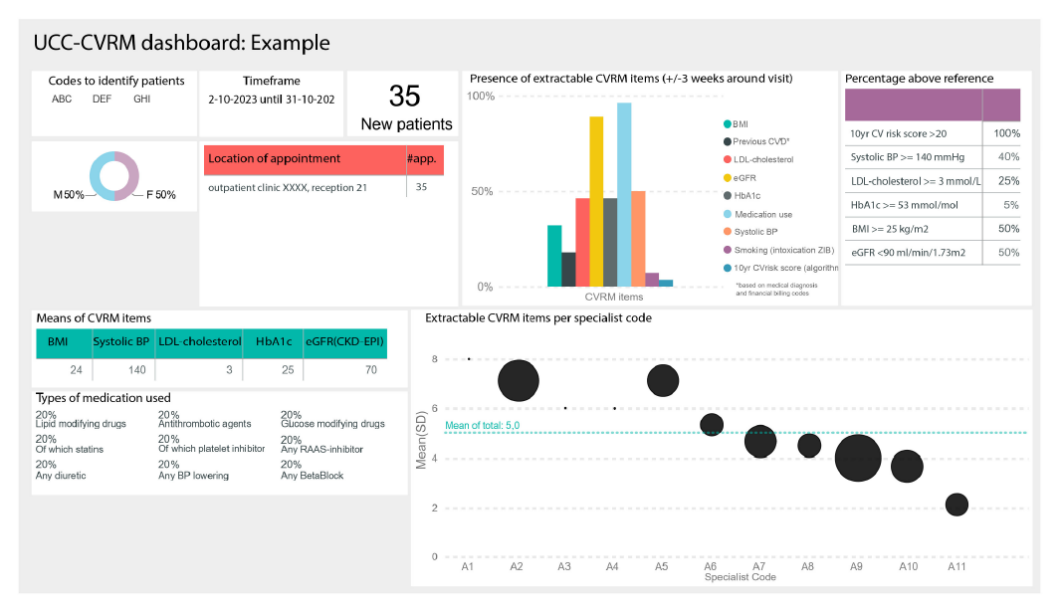
Example of a UCC-CVRM dashboard on extractable CVRM information from the EHR [11]. BP: blood pressure; CKD-EPI: Chronic Kidney Disease Epidemiology; CVD: cardiovascular disease; CVRM: cardiovascular risk management; eGFR: estimated glomerular filtration rate; HbA_1c_: glycated hemoglobin; LDL: low-density lipoprotein; UCC-CVRM: Utrecht Cardiovascular Cohort-Cardiovascular Risk Management.

All contact persons receiving the dashboards (ie, the clinicians involved in the development of the dashboards) also received information on the specific locations in the EHR from which the indicators were extracted, as presented in Table S1 in Multimedia Appendix 1. We sent the dashboards on a regular basis (ie, monthly or every three months, depending on the preference of the department) to clinicians of the participating departments who were appointed as contact persons for UCC-CVRM activities (Table S2 in Multimedia Appendix 2). The dashboards included information on (1) the codes used to identify the subset of patients to which CVRM guidelines apply, (2) the time frame indicating the period when the patients visited the UMC Utrecht, (3) the total number of patients included in this dashboard during the specified time frame, (4) a bar chart showing the percentage of patients for whom the 8 CVRM indicators could be extracted (so the 10-y cardiovascular risk could be calculated), (5) the percentage of patients who had values above the reference value for 6 indicators, (6) sex distribution of the patients included in the dashboard, (7) the location of the appointment in the hospital, (8) the distributions of CVRM indicators, (9) the percentage of patients using prespecified types of medication, and (10) the average number of extractable items per treating clinician (Figure 2) [11]. Pseudonymized clinician codes were presented on the x-axis of this item, so that individual treating clinicians were not aware of their own nor their colleagues’ code. Small differences in the content were made depending on the preferences of the department (Table S2 in Multimedia Appendix 2).

### Primary and Secondary Outcomes

The primary outcome of the quantitative part of this study was the extractability of the CVRM indicators and whether the extractability changed over time after the implementation of the dashboards. Indicators were considered extractable if the value could be extracted from a predefined structured field of the EHR. To note, if performed locally in our hospital, laboratory values are automatically registered in the designated structured fields of the EHR. Thus, if a laboratory value was not extractable, no laboratory measurement was done locally in the UMC Utrecht. The other CVRM indicators can be registered in structured fields of the EHR or other fields, such as clinical notes. Thus, if these indicators were not extractable, the indicator was either not measured or was registered in a field from which the information is not extractable (eg, clinical notes).

The outcomes of the qualitative part were the perceptions, views, and explanations of clinicians related to the information in the Power BI dashboards, assessed by means of the semistructured interviews.

### Phase 1: Extractability of CVRM Indicators Over Time

#### Setting

The data were collected using the Utrecht Patient Oriented Database (UPOD) of the UMC Utrecht. The UPOD contains all data registered in hospital systems of all patients who have visited the hospital since 2004. More detailed information on the UPOD has been published elsewhere [12]. All patients who visited a participating department of the LHS at the UMC Utrecht for a first-time evaluation of a cardiovascular risk factor or CVD between January 2022 and November 2023 were eligible for participation. The inclusion criteria for this study consisted of being an adult (aged ≥18 y) attending the UMC Utrecht for a first-time evaluation of a CVD or CVD risk factor. In close collaboration with clinicians from the specific departments, the eligible patients were identified based on appointment and agenda codes as registered in the EHR, or, in the case of the neurology and vascular surgery department, based on diagnosis billing codes (DBC). Furthermore, the patients should have had a first visit to the cardiology, vascular surgery, vascular medicine, geriatrics, neurology, nephrology, or the diabetology department. Patients who visited the nephrology outpatient department with a prior kidney transplantation within 365 days of their appointment were excluded, as these patients were not considered eligible for CVRM.

#### Data Collection

The date of the new appointment was used as index date for the additional data collection. For the vascular surgery patients, the date of the new appointment closest to the DBC date was used as index. The date of the DBC was used as an index for the neurology department. We collected demographic data (ie, age and sex at birth) from structured fields of the EHR of all patients. Age was calculated by subtracting the index date from the date of birth. Additionally, we collected data on physical measurements (ie, height, weight, and BMI), laboratory measurements (ie, total cholesterol, high-density lipoprotein and low-density lipoprotein cholesterol, and triglycerides), glycated hemoglobin and hemoglobin, and smoking status from structured fields of the EHR. The values closest to the index date, but within a window of 21 days, were extracted. Renal function was determined using the estimated glomerular filtration rate (eGFR), calculated using the Chronic Kidney Disease Epidemiology Collaboration equation [13]. Systolic and diastolic blood pressure measurements recorded in structured fields of the EHR within 7 days of the index date were extracted. Finally, we collected information from the EHR regarding medication use and history of CVD. The presence of a CVD history, which included stroke, peripheral artery disease, coronary heart disease, and abdominal aortic aneurysm, was determined using an in-hospital developed and validated algorithm based on billing codes, hospital procedures, and medical diagnoses registered in the EHR before the index date. The 10-year risk score for CVD morbidity or mortality was calculated using the SCORE-NL [14].

#### Data Analysis

**Figure 3.**
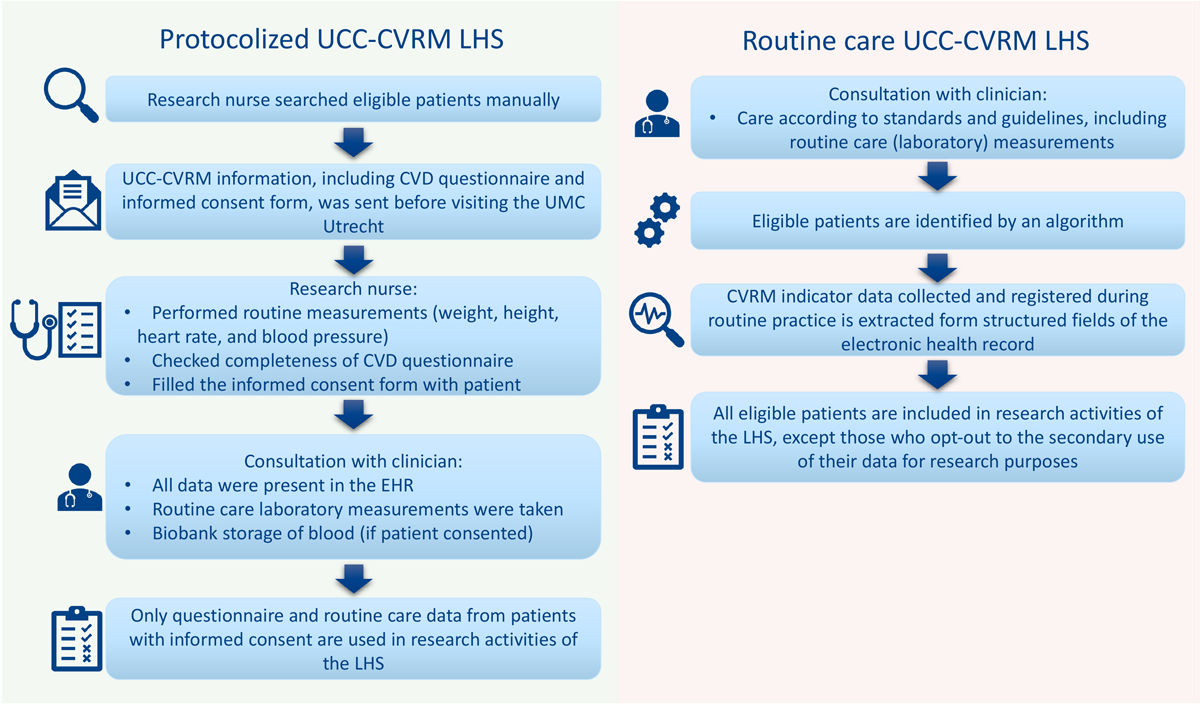
Patient inclusion and data collection procedure of the protocolized LHS and the current LHS fully digitalized and based on routine practice. Adapted from Zondag et al [16]. CVD: cardiovascular disease; CVRM: cardiovascular risk management; EHR: electronic health record; LHS: learning health care system; UCC-CVRM: Utrecht Cardiovascular Cohort Cardiovascular Risk Management; UMC: university medical center.

We described the characteristics of the study population as means and SDs, medians with corresponding first and third quartiles, or as numbers and percentages, as appropriate. Then, we presented the percentage of extractable indicators for the whole study period (ie, January 2022 to November 2023) and stratified by pseudonymized department. Additionally, the extractability of the individual indicators was assessed over time. Next, we calculated the mean number of extractable indicators per patient (max 7 CVRM items: systolic blood pressure, BMI, hemoglobin, smoking status, low-density lipoprotein cholesterol, glycated hemoglobin, and eGFR) per 6 months, stratified by sex and department, and visually assessed trends. Finally, when the patient inclusion procedure for the cardiovascular LHS was done manually and performing measurements was systematically supported and protocolized (Figure 3), Groenhof et al [2] compared the extractability of the CVRM indicators with the extractability before the initiation of the UCC-CVRM LHS. We compared our results to theirs by comparing the extractability before the UCC-CVRM initiation and the protocolized UCC-CVRM with the UCC-CVRM extractability of indicators based on routine care (this study).

R version 4.0.5 was used for all quantitative analyses [15].

### Phase 2: Experiences and Perspectives of Clinicians on the Dashboards

#### Data Collection

We collected qualitative data via semistructured interviews. A semistructured topic list with predefined questions was constructed, including topics regarding the value of such dashboards for patients and clinicians and the clinician’s experience with the dashboards (Tables S3 and S4 in Multimedia Appendix 3). We used a purposive sampling method; we approached those with experience with the dashboard and included those willing and able to participate in the study. We invited clinicians working at departments who regularly received the UCC-CVRM dashboards for an interview via email. AGMZ, a qualified female researcher, conducted the interviews in person at the UMC Utrecht between June 2024 and July 2024. Directly after each interview, AGMZ summarized the interview and made field notes, including the most important and surprising themes that came up during the interviews. *Important* themes referred to issues that were frequently emphasized or emotionally charged, whereas *surprising* themes referred to insights or experiences that were unexpected considering the research question or existing literature. The purpose of noting these was 2-fold: (1) to begin reflexively engaging with the data early in the process and (2) to help sensitize the formal coding phase to patterns that might otherwise be overlooked. Each interview was audio recorded, transcribed verbatim, and anonymized by a professional transcription service. AGMZ checked the transcripts for reliability. The interviews were conducted in Dutch, and informative quotes were translated into English by the research team. We additionally collected demographic data of the respondents (ie, age, sex, and job title). Our sampling approach was guided by the principle of coding saturation, that is, until no new codes or themes emerged from the interview data.

#### Data Analysis

The transcripts were analyzed thematically using inductive and deductive coding. Based on the topic list, AGMZ developed an initial set of codes. NVivo (version 12) was used to code the interviews [17]. After coding 3 transcripts, AGMZ began grouping several codes into themes and subthemes, which resulted in a first code tree. To ensure quality and rigor, the full research team was involved in discussing and refining the coding framework, reviewing initial themes, and resolving discrepancies. These collaborative sessions allowed for reflection on possible biases, ensured consistency in interpretation, and enhanced the confirmability and credibility of the findings. Then, the remaining transcripts were coded, and the code tree was adjusted if necessary. Finally, relevant quotes were selected to illustrate the identified themes and subthemes.

### Ethical Considerations

The study protocol was submitted to the Research Ethics Committee of the UMC Utrecht for review (23U-0181) before the initiation of both phase 1 and phase 2 of this study. The ethics committee determined that this study was exempt from the Dutch Medical Research Involving Human Subjects Act (WMO). The UMC Utrecht carried out an independent quality check to ensure compliance with legislation and regulations. Patients who objected to the (re-)use of their data for scientific research by indicating their opt-out in the UMC Utrecht’s patient portal were excluded from phase 1. For phase 2 of the study, we obtained written informed consent from all respondents before the interview. The participants in this study did not receive a compensation for their participation.

We used the COREQ (Consolidated criteria for Reporting Qualitative research) checklist as a guideline to report our qualitative research results.

## Results

### Phase 1: Extractability of CVRM Indicators Over Time

#### Patient Characteristics

Between January 1, 2022, and November 1, 2023, a total of 8941 first-time visits took place at the participating CVRM departments of the UMC Utrecht (Table 1). The median age of the patients was 61 (IQR 45‐72) years, and 49.9% (4466/8941) were women. The median 10-year risk score for CVD morbidity and mortality was 22% (IQR 7.0%‐35.0%). Characteristics of the patients stratified by department are presented in Table S5 in Multimedia Appendix 4.

**Table 1.** Patient characteristics based on data extracted from structured fields of the electronic health record.

	Total (N=8941)
Age, median (IQR)	61.0 (45.0‐72.0)
Female, n (%)	4466 (49.9)
Smoker, n (%)	445 (17.5)
BMI (kg/m^2^), mean (SD)	26.5 (5.1)
Systolic blood pressure (mm Hg), mean (SD)	143.7 (28.0)
LDL^a^ cholesterol (mmol/L), median (IQR)	2.5 (1.9‐3.3)
Triglycerides (mmol/L), median (IQR)	1.4 (1.0‐2.1)
HbA_1c_^b^ (mmol/mol), median (IQR)	38.0 (35.0‐44.0)
Hemoglobin (mmol/L), mean (SD)	8.4 (1.2)
Creatinine (µmol/L), mean (SD)	90.0 (75.0)
eGFR CKD-EPI^c^ (mL/min/1.73m^2^), mean (SD)	82.9 (28.6)
CVD^d^ history (yes), n (%)	1871 (95.5)
SCORE-NL (%), median (IQR)	22.0 (7.0‐35.0)

a LDL: low-density lipoprotein.

b HbA_1c_: glycated hemoglobin.

c eGFR CKD-EPI: estimated glomerular filtration rate using the Chronic Kidney Disease Epidemiology Collaboration equation [13].

d CVD: cardiovascular disease.

#### Presence of Extractable CVRM Indicators in EHRs

**Figure 4.**
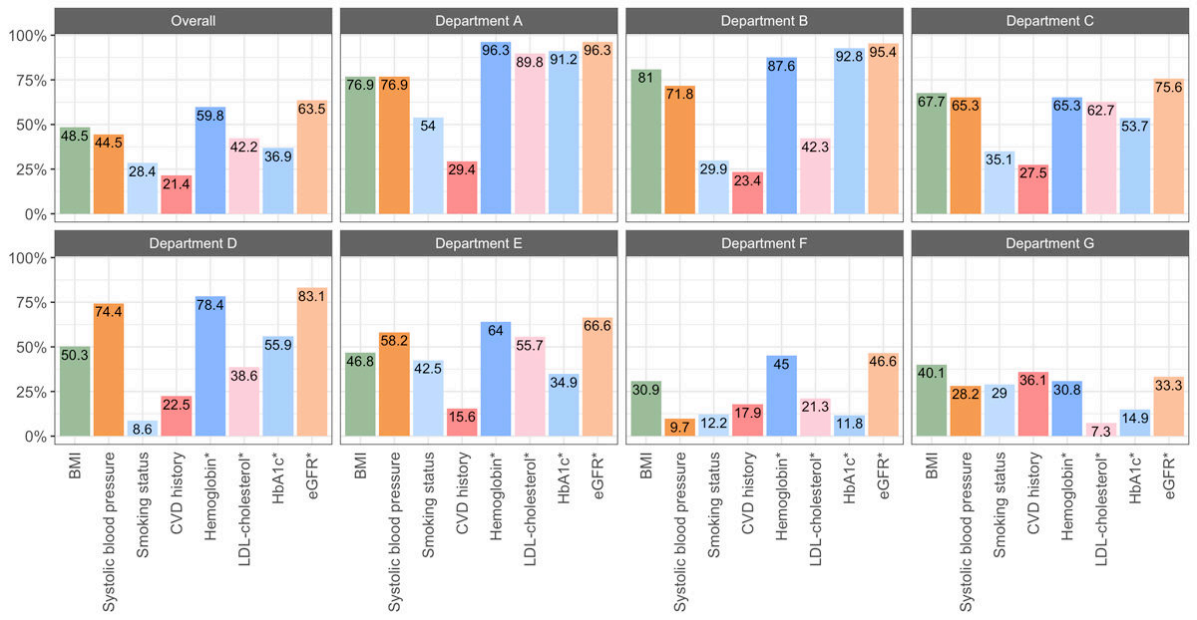
Extractability of cardiovascular risk management indicators from structured fields of the electronic health record (EHR) in percentages, in the total population and by department. *If this indicator was not extractable, no measurement was performed locally in the hospital, as laboratory results are automatically registered in the designated fixed structured field of the EHR and thus extractable. CVD: cardiovascular disease; LDL: low-density lipoprotein; eGFR: estimated glomerular filtration rate; HbA_1c_: glycated hemoglobin.

**Figure 5.**
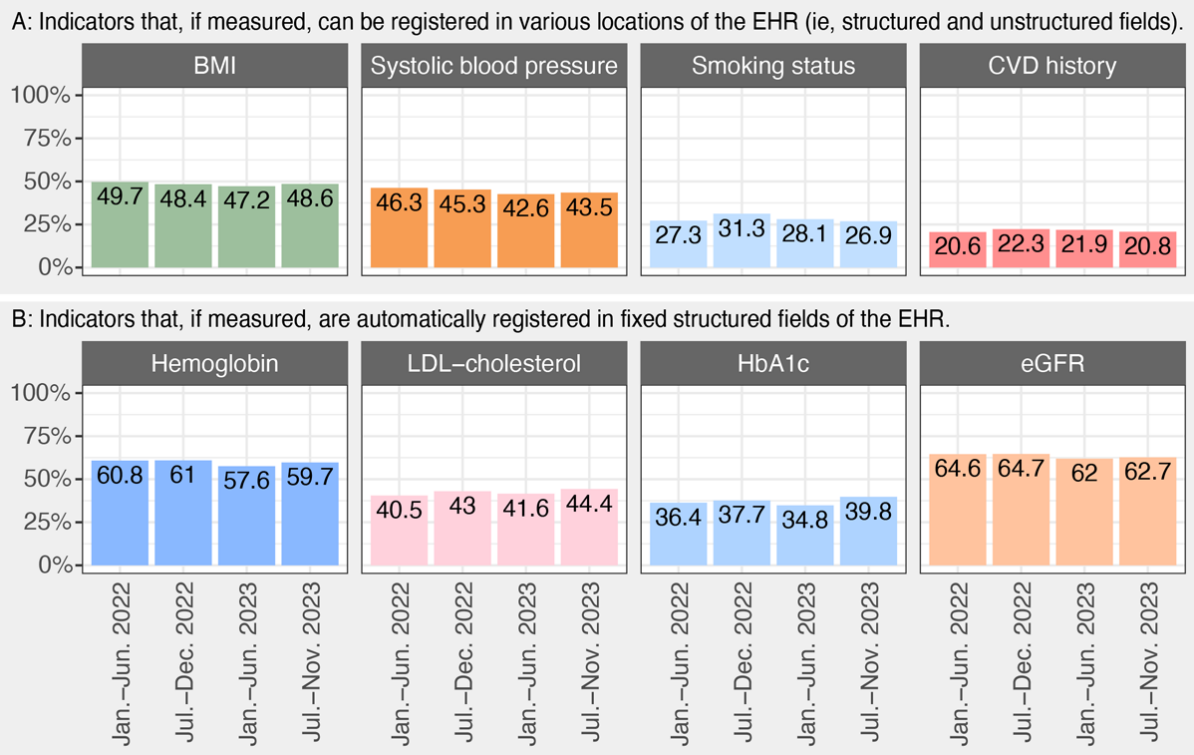
Overall extractability over time, per cardiovascular risk management indicator. Panel A illustrates indicators that may be registered in other locations in the EHR than the structured fields, and Panel B illustrates indicators that are always extractable if measured. CVD: cardiovascular disease; eGFR: estimated glomerular filtration rate; EHR: electronic health record; HbA1c: glycated hemoglobin; LDL: low-density lipoprotein.

Overall, hemoglobin (5344/8941, 59.8%) and eGFR (5682/8941, 63.5%) were most often registered in structured fields of the EHR, and the patient’s CVD history (1946/8941, 21.4%) and smoking status (2543/8941, 28.4%) were registered the least frequently, yet the extractability of the indicators varied between departments (Figure 4). The extractability of these CVRM indicators did not improve over the period in which the dashboards were implemented (ie, January 2022 to November 2023; Figure 5).

#### Mean Extractable CVRM Indicators Per Patient

**Figure 6.**
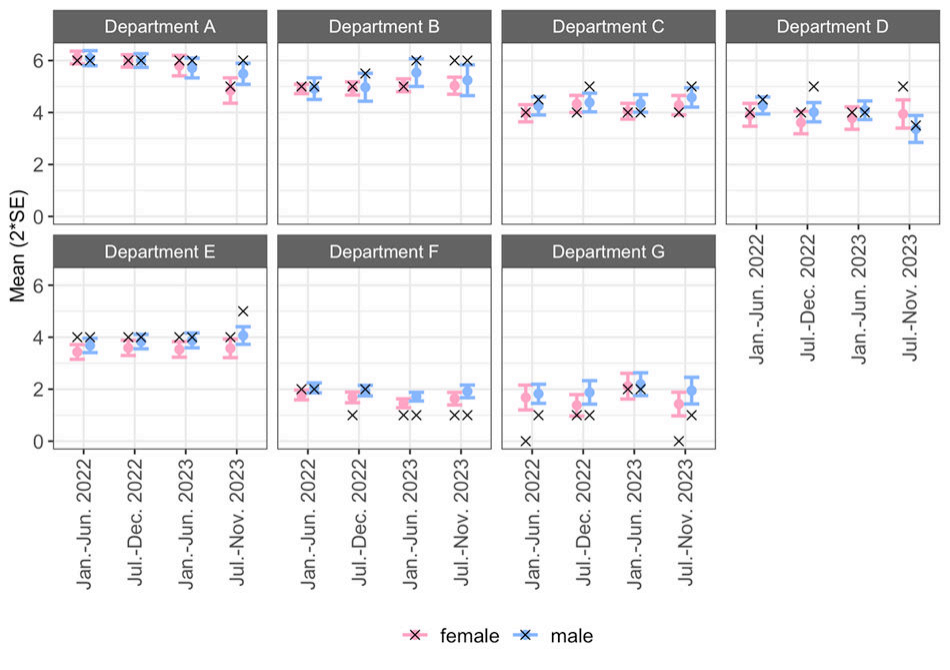
Mean number of extractable CVRM indicators over time (maximum is 7 indicators), by department and sex. x: median number of extractable CVRM indicators. CVRM: cardiovascular risk management

Extractability of the cardiovascular risk profile was the lowest in departments F and G, where a mean of 1.8 (SD 1.9 and 2.1, respectively) of 7 CVRM indicators could be extracted per patient (Figure 6). Departments A and B scored the highest in terms of the number of indicators registered in structured fields of the EHR, with a mean of 5.8 (SD 1.3) to 5.0 (SD 1.3) of 7 CVRM indicators. Trends in extractability did not visibly change over time and were similar between sexes.

#### Comparing Before UCC-CVRM, Protocolized UCC-CVRM, and Digitalized UCC-CVRM

At the time the UCC-CVRM was initiated, the extractability of physical and laboratory measurements significantly increased compared to the situation before the initiation of the UCC-CVRM (extractability between 25% and 77% vs >80%, respectively; Table 2) [2]. When the extraction of CVRM indicators was digitalized and the protocolized measurement of indicators was discontinued, the extractability of the indicators nearly reduced to the levels of ‘before UCC-CVRM’. The extractability of glycated hemoglobin decreased by 45%, from 82% to 37%. While still substantially lower than in the 2 previous periods (64% vs 76%‐87%), the extractability of eGFR remained the most stable between the 3 periods. In the current ‘routine care UCC-CVRM’ (≥2022), we did not observe the sex difference that was present in the ‘before UCC-CVRM’ group (<2015).

**Table 2.** Comparison of the extractability of cardiovascular risk management indicators, in total and by sex.

Indicator	Before UCC-CVRM^a^			Protocolized UCC-CVRM			Routine care UCC-CVRM		
	Total (n=7195)	Male (n=3685)	Female (n=3510)	Total (n=1904)	Male (n=974)	Female (n=930)	Total (n=8941)	Male (n=4475)	Female (n=4466)
BMI (%)	57	63	52	93	94	91	49	48	49
SYSBP^b^ (%)	77	82	72	93	94	91	45	43	46
LDL-C^c^ (%)	33	35	32	83	79	87	42	45	41
eGFR^d^ (%)	76	82	70	87	87	87	64	64	62
HbA_1c_^e^ (%)	25	28	22	82	78	85	37	36	37

a UCC-CVRM: Utrecht Cardiovascular Cohort-Cardiovascular Risk Management.

b SYSBP: systolic blood pressure.

c LDL-C: low-density lipoprotein cholesterol.

d eGFR: estimated glomerular filtration rate using the Chronic Kidney Disease Epidemiology Collaboration equation [13].

e HbA_1c_: glycated hemoglobin.

### Phase 2: Experiences and Perspectives of Clinicians on the Dashboards

#### Demographics

Of the 9 clinicians approached for an interview, 5 (56%) agreed to participate in the study, 3 (33%) did not respond, and 1 (11%) responded only after the possible time frame of the interviews. The interviews took place between June 17 and July 30, 2024. The median duration of the interviews was 21.4 (IQR 19.1‐22.1) minutes. On average, the respondents were aged 48 (SD 8.5) years, 80% (4/5) were men, and the majority (4/5, 80%) were familiar with the dashboards (Table 3).

**Table 3.** Demographics of the respondents.

Characteristic	Respondents (n=5)
Sex, n (%)	
Male	4 (80)
Female	1 (20)
Age, mean (SD)	48 (8.5)
Experience with the dashboards, n (%)	
Yes	4 (80)
No	1 (20)
Job title, n (%)	
Cardiologist	1 (20)
Nephrologist	1 (20)
Vascular surgeon	1 (20)
Internist vascular medicine	1 (20)
Neurologist	1 (20)

Overall, the respondents were positive about the dashboards and referred to their benefits. However, they argued that implementing and mobilizing colleagues to improve the extractability of CVRM indicators from structured fields is challenging. The clinicians’ views and perceptions about the dashboards and the unchanged extractability of CVRM indicators over time were clustered around 3 themes: (1) challenges at the organizational level hampering structured registration of CVRM indicators, (2) technical difficulties mainly related to the EHR system used in the hospital, and (3) issues at a more operational level. Potential solutions were also provided by the clinicians related to improving the extractability and the use of the dashboards and were equally clustered around these themes.

### Theme 1: Organizational Level

#### Responsibility for CVRM

Several respondents emphasized that having a comprehensive picture of their patient’s CVD risk profile is necessary to ensure quality of care and safety. Furthermore, they stressed that because they work in an academic hospital, they felt responsible to provide care in such a way that the information collected during clinical practice can be reused for research purposes (Table 4, Quote 1A). Therefore, they argued it to be their responsibility to assess the CVRM indicators and to register the indicators in structured fields of the EHR. Nevertheless, they also mentioned that not all colleagues of their department felt the same way about this. For example, their colleagues frequently mentioned not needing the complete CVD profile to provide the best care for their patient (Table 4, Quote 1B). Diabetes and cholesterol were, for example, not considered relevant risk factors for some of their patients. Yet, some respondents did not necessarily see CVRM as their primary responsibility and felt that CVRM is the responsibility of other medical professionals, such as general practitioners or colleagues from internal medicine (Table 4, Quote 1C). It was suggested that, if the full CVD profile is to be assessed and documented in designated fields of the EHR during the first appointment of the patients, this task and responsibility should be assigned to a specific health care provider (eg, outpatient clinic assistant). Additionally, respondents mentioned the lack of concrete agreements about how and where to register information in the EHR as a reason for the variability in the registration (Table 4, Quote 1D). It was suggested that a discussion to realize a more systematic and protocolized way of providing care would be necessary to improve the quality of registration (Table 4, Quote 1E). However, it was argued that specialists may not sustainably change their registration habits, even if complete registration of CVRM indicators were the responsibility of the specialist.

**Table 4.** Quotes illustrating theme 1: the organizational hurdles.

Subtheme	Illustrative quote
Responsibility for CVRM	
1A	R4: “My personal opinion is that [clinical department] is clearly a clinical field, but it is also very much a research field. I think that we, as [name job function], should be at the forefront of this and that is also what I have tried to explain to colleagues.”
1B	R1: “That [discussing the need of complete and uniform CVRM to colleagues] resulted in predictable reactions. For several things [CVRM indicators], their reaction was: ‘Yes, but I do not need it for the care I provide’.”
1C	R2: “The reason why we did not implement this in our department is that, [...] we think that a lot of this, as customary, simply belongs to the general practitioner.”
1D	R2: “As long as we don’t have a consensus on how we’re going to register... then it’s never going to happen.”
1E	R4: “...working in a protocolized manner at an outpatient clinic can contribute greatly to the quality and also the safety of the care you provide. So, I am very much in favor of a checklist, so to speak, and protocolized care pathways.”

### Theme 2: Technical Level

Many of the respondents’ perceptions that explained the suboptimal extractability of CVRM risk factors relate to issues at a more technical level. These technical reasons are grouped into the following themes: the usability and familiarity of the EHR and the perceived incompleteness of the dashboards due to the data fields in the EHR used for data extraction.

#### Usability and Familiarity of the EHR System

Most of the respondents regarded the EHR system to be user unfriendly and an important explanation for why extractability may have been suboptimal. Some of the structured fields, for example, smoking status, were not easily accessible in the EHR, as they required opening multiple tabs of the patient’s health record (Table 5, Quote 2A). Some respondents mentioned that, at times, the knowledge of some of their colleagues about the functionalities of the EHR system and how to make efficient use of these functionalities seemed to be lacking (Table 5, Quote 2B). Another reason for not always using the structured fields of the EHR for the registration of CVRM indicators was that, for some items, the information documented in structured fields is not automatically included in the referral letters generated by the EHR system, whereas the information in unstructured fields is (Table 5, Quote 2C).

**Table 5.** Quotes illustrating theme 2: the technical difficulties.

Subtheme	Illustrative quote
Usability of the EHR^a^ system	
2A	R1: “There were things not well organized [in the EHR system]. And because of that, the registration… smoking for example, that field was just too hidden.”
2B	R4: “I think that not everyone is equally adept with the EHR system, and that not everyone has the same discipline to use it as it is intended. Because of that, people sometimes experience it as an administrative burden to have to put things in a certain place, while in fact, if you were to apply it [the functionalities], it would actually promote more efficiency.”
2C	R3: “What really is stupid, for example, for the medical history there is such a standard item [in the EHR system]. If you fill it in there, it doesn’t appear in your letter. So that’s why we put it in the anamnesis field, where it’s not really intended for.”
2D	R3: “That you have a standard anamnesis field, which includes, for example, the standard intoxication questions. Then you already see them and you only have to click to answer the question ...”
Incompleteness of the dashboard	
2E	R2: “The tone was immediately that we were not going to implement this, because it’s just not complete.”
2F	R5: “... everything has to be in there [in the dashboard] that can be retrieved from the system. And then those free text fields are also part of it, but I am aware that, in terms of searching for this information, it is undoubtedly a difficult and challenging exercise.”

a EHR: electronic health record.

A potential solution mentioned was to add a function enabling the creation of a template for the anamnesis page in the EHR, including all structured fields from which the data are extracted for the dashboard (Table 5, Quote 2D). According to the respondents, the recent update of the EHR in March 2024 potentially improved the structured registration of indicators as, from their perspective, fields are easier to access and templates can be made per department, improving the opportunity for uniform registration.

#### Incompleteness of the Dashboard

Some respondents considered the information presented in the dashboards to be incomplete, that is, lacking the information documented in unstructured fields and other data sources (eg, referral letters). This was named as a reason for not implementing the dashboards in their department, and therefore, the extractability was and remained low over time (Table 5, Quote 2E). The current data collection is perceived as incomplete by some respondents because a lot of information could probably be extracted from other fields of the EHR (ie, free text) or by linking to other data sources, such as primary care databases. Respondents mentioned that it would be more beneficial for the LHS if we extracted the data from all possible fields of the EHR, although they did acknowledge that this would probably be very challenging (Table 5, Quote 2F). Instead, efficient use of UMC Utrecht’s patient portal was mentioned as potentially helpful for the collection of certain indicators that could be documented by patients themselves before their appointments. A link between the patient portal and the EHR system would then be needed to integrate the information from the patient portal into the designated structured fields in the EHR.

### Theme 3: Operational Level

Several operational reasons were mentioned that could explain the extractability of indicators remaining unchanged since the implementation of the dashboards. These relate to the following themes: time constraints and the habitual work practices of health care providers.

#### Time Constraints

Multiple respondents stated that time constraints may often be the reason why indicators are often registered in the unstructured fields of the EHR. Lack of time was partly due to the limited amount of time per consultation (ie, 10‐15 min for a follow-up consultation and 30‐45 min for intake of a new patient, depending on the department and specialty) and because the administrative burden is already considered high (Table 6, Quote 3A). Therefore, it is perceived to be easier and quicker to record the information in unstructured fields of the EHR, resulting in indicators not being extractable. Finally, it was said that when the dashboards were implemented, the focus was on improving the extractability of ‘easier to influence’ indicators (ie, physical and laboratory measurements), which explains the low extractability of the ‘harder to influence’ indicators, such as smoking. When starting a conversation about smoking and the patient happens to smoke, it would mean that a more detailed conversation about smoking and quitting should follow (Table 6, Quote 3B). It was argued that this would result in a lack of time because such a conversation would take as much time as planned for the full consultation, leaving little to no time to assess the other risk factors.

**Table 6.** Quotes illustrating theme 3: the issues on an operational level.

Subtheme	Illustrative quote
Time constraints	
3A	R2: “You have a limited amount of time... And, naturally, you cannot do everything.”
3B	R1: “A really good conversation about smoking is, in the context of a technique like motivational interviewing or something like that, really a time-consuming job. If you then have a consultation during consultation hour in which you must discuss seven risk factors... and you then think about starting the conversation about smoking, then your consultation hour is over, and you haven’t addressed the other six risk factors yet.”
3C	R5: “I think that a lot of those risk factors, if they would have been already pre-filled by the patient or the outpatient assistant... that it would already help. I mean, that would save us five minutes time in a consultation.”
Individual habits	
3D	R1: “There is simply a group of people who are super persistent to, if they measured blood pressure at all, document this in the free text of the EHR and did not have the decency to put it in the standardized field designed for that purpose.”
3E	R4: “I think it’s much more about showing that if you work in a structured way, it’s more efficient. That’s, what I think, my colleagues are looking for. Especially: how can I do my consultations in a structured and efficient way? When correct administration contributes to efficiency, safety, and effectiveness of the care provided... then people are, I think, prepared to work with that. Because what they find important is providing efficient, safe and effective care. So, when you can combine those goals, so that structured recording of certain patient data in the EHR contributes to achieving those goals, and thus supports the work process of the healthcare provider... then you will get them to cooperate. I expect that, ultimately, that is the way to ensure that care providers embrace that work process...”
3F	R1: “I do believe in a kind of ‘name and shame’, or ‘name and improve’ culture. I really think that there are people... well, I would never want to be the worst in the class myself. I don’t have to be the best, but not being the worst is definitely my goal.”

It was mentioned that using the patient portal could alleviate time constraints by enabling patients to document certain indicators (eg, their smoking status, height, and weight) themselves before their visit. This would enable health care providers to allocate more time to assess other indicators that patients cannot assess and document themselves, such as laboratory data. Other considerations to reduce time pressure included delegating CVRM as a task for the nurse specialist or outpatient assistant (Table 6, Quote 3C).

#### Individual Habits

Another challenge in improving extractability was, according to respondents, related to the habits of the health care providers. Habits of documenting information in the EHR were considered difficult to change (Table 6, Quote 3D). It was said that not all colleagues seemed to regard structured and uniform registration of risk factors as beneficial, and the majority considered these burdensome administrative tasks and, therefore, were not willing to change their habits. Convincing health care providers that structured registration leads to more efficient, safe, and effective care could alter this unwillingness, as this is in line with what is important to the health care providers (Table 6, Quote 3E). Another frequently mentioned suggestion for motivating behavior change and thus improving extractability was peer comparison. Respondents emphasized that being able to compare their ‘score’ to anonymous other individuals or departments would motivate them to perform better in terms of documenting indicators in designated fields of the EHR (Table 6, Quote 3F).

## Discussion

### Principal Findings

This study aimed to evaluate whether the implementation of dashboards in our LHS improved the structured registration of CVRM indicators in patients with an increased risk of CVD. The extractability of the indicators was low, and although the extractability varied between departments, we did not observe substantial changes over the period during which the dashboards were shared with the departments. From that standpoint, the dashboards have not been effective. While in-depth semistructured interviews revealed that respondents considered such dashboards as useful, disproportionate burdens were perceived by the respondents themselves or their colleagues, explaining the low and unchanged extractability of the CVRM indicators. The registration of the indicators was hindered due to organizational (ie, discussions on who was responsible for CVRM and the lack of agreements surrounding the registration), technical (ie, usability and technical knowledge of the EHR system and its functionalities, incompleteness of the dashboard), and operational issues (ie, time constraints and individual habits). Potential ways to improve the extractability included making use of the patient portal, using peer comparison, and educating about the clinical benefits of structured registration.

### Comparison With Existing Literature

Several reviews evaluated the use and impact of dashboards in a health care setting by assessing, among others, before-after studies and randomized controlled trials (RCTs) [5,6,18]. However, the settings, designs, and aims of the studies included were highly heterogeneous, and the results of the systematic reviews are inconclusive [5,6,18]. The findings suggest that dashboard effectiveness may depend on clinical context, patient population, theoretical underpinning, and specific implementation features. So far, none of the studies have focused on the extractability or structured registration of CVRM indicators as the outcome of interest. Still, one of the included RCTs, a parallel arm cluster RCT, showed that a computer-guided intervention, which included decision support, audit and feedback tools, and training, improved cardiovascular risk factor screening. The impact of the intervention was compared to usual care (ie, to sites not randomized to the intervention and, thus, did not have access to the intervention tool) [19].

Respondents in our study acknowledged that recording CVRM indicators in a structured manner is valuable for clinical care and secondary use of the data, yet not all care providers seemed to support and adopt structured registration in practice. This is in line with previous research [20]. The reasons provided by the respondents of our study are echoed in other studies [21-23]. For example, Marani et al [21] compared the availability of data in structured fields of the EHR with manual chart review in diabetes patients and found that the availability of data extracted from structured fields was much lower than when the information was manually searched for in EHRs [21]. While we only extracted data from structured fields and, thus, cannot be certain that the CVRM indicators were in fact present in other parts of the EHR, it is highly likely that the information would indeed be more complete had we included unstructured sections of the EHR, an issue also mentioned by the respondents. However, to support clinical decision-making, improve patient care, and validly reuse routine care data for research and other secondary activities, routine care data should at least equal the quality of the information registered currently in the EHR. This can only happen when information is stored in a structured and standardized format in the EHR [20,23,24]. It is, therefore, desirable to improve the registration in structured fields of the EHR, through systematically creating awareness and educating medical students, specialists in training, and the present medical staff.

As mentioned, the low extractability of CVRM indicators was partly explained by the perceived organizational issues. Some clinicians did not seem to feel responsible for CVRM and felt that agreements surrounding registration are lacking. This is supported by other studies that assessed the care providers’ attitudes toward structured problem lists in the EHR [23,25]. One study found that general disagreement between specialists and primary care practitioners about who is responsible for the problem list was a major cause of the gaps in the problem lists and mentioned the need for guidelines and policies to clarify this [25]. From a technical standpoint, the user interface of the EHR was seen as a barrier to structured registration, especially for indicators that required opening additional tabs on top of the main tab. In March 2024, however, the UMC Utrecht updated its EHR system, which now has the possibility of creating customized templates that could include all fields that are deemed as necessary to complete. Respondents acknowledged that this update is expected to improve the extractability of CVRM indicators in the long term. Future studies are necessary to explore this expectation in our hospital.

### Implications and Recommendations

Our findings indicate that there are underlying barriers to structured registration that need to be addressed and suggest that educational efforts are needed to improve care providers’ knowledge on the EHR system functionalities and the benefits of structured registration of CVRM indicators. Educational efforts have been found to be the most efficient and effective interventions to improve structured registration of routine care data in EHR records [26]. During these efforts, it is important to emphasize the direct benefit of structured registration for the care provider, rather than focusing on the secondary benefit (ie, research). Benefits for the care provider include that using a structured registration leads to better and faster decision-making [27], and that it enables the use of CVD algorithms used for clinical decision support. These clinical decision support systems (CDSSs) oftentimes require a standardized set of data to assess the patient risk and treatment possibilities [28]. These CDSSs can be linked to the EHR and automatically load the EHR data into the CDSS to provide a risk prediction during routine clinical practice [29], removing the need for manual entry, which will likely motivate care providers to pay more attention to structured and systematic registration of CVRM indicators during clinical practice. In addition, technical training on how to use all functionalities of the EHR system seems equally necessary, as it was mentioned by the respondents that not all health care providers are equally adept with the EHR system, hampering efficiency. Besides, to reduce the administrative burden on care providers, it is useful to explore the use of the hospital’s patient portal for the collection of CVRM indicators that can easily be assessed and documented by patients themselves, such as blood pressure. The portal should be linked to the EHR, and the values automatically loaded into the predefined structured fields of the EHR.

Furthermore, as mentioned by others [23,26,30], clarification and policies regarding responsibilities for structured registration and harmonization of items to be recorded are necessary. Moreover, behavioral change must be realized to gain the benefits of these dashboards. Peer comparison and allowing departments to compare their performance with other departments can be an effective strategy, as mentioned by the respondents in this study, and has proven to be successful in previous research [30-32]. While a peer comparison component was already present in our dashboards (ie, a figure showing the average number of CVRM indicators recorded in structured fields per clinician), pseudonymization of all names obscured such comparison and may have led the component to be less effective. Clinicians are expected to become more motivated to change their registration practices once we make known which code belongs to them, whereas their colleagues’ codes remain anonymous to them.

### Strengths and Limitations

To our knowledge, we are among the first to report on a mixed methods study to evaluate the implementation of dashboards for feedback purposes to clinicians. The mixed methods design allows for combining the strengths that are inherent to the individual qualitative and quantitative approaches and to mitigate the weaknesses [33]. While there are various mixed methods study designs, we opted for the sequential explanatory design, in which the quantitative data collection and analysis precedes the qualitative part. The benefit of using this design is that it allowed for a thorough explanation of our quantitative results, as we were able to design the qualitative phase based on the quantitative results [33].

Our study has several limitations that warrant careful interpretation of the results. Regarding the comparison between the ‘before UCC-CVRM’, ‘protocolized UCC-CVRM’, and ‘routine care UCC-CVRM’, it is important to note that the time window of the data collection in the ‘before UCC-CVRM’ is somewhat different than the time window of the ‘routine care UCC-CVRM’ [2], which may explain some of the differences in the extractability between the two, making them somewhat less comparable. Besides, the extractability of the CVRM indicators in the ‘protocolized UCC-CVRM’ is based on patients who provided explicit consent only, as an informed consent procedure was in place at that time. The extractability of the CVRM indicators in patients without consent was lower [34,35].

In addition, given that this is a single-center study, the generalizability of the findings to other settings may be limited. Further research in different settings is needed to validate our results. Furthermore, although coding saturation was achieved, the relatively limited sample of interviewed clinicians, along with the underrepresentation of female clinicians among the interviewees, may have limited the diversity of perspectives and might not fully represent the views of all health care professionals or all clinical departments. A larger, more diverse sample might provide additional perspectives on the perceived barriers.

Furthermore, the results of our study may be influenced by (self-)selection bias. The majority of the respondents who were invited to participate in an interview were the primary contact persons within the cardiovascular LHS for such initiatives. Those who volunteered to participate in our study might have had particularly strong views or experiences related to the topic.

Next, the respondents work in an academic hospital and are therefore expected to actively participate in clinical research and implementation initiatives, which may have led to social desirability bias. However, we believe that this bias was limited as we took several measures to minimize the risk of social desirability bias. First, we used semistructured interviews as the data collection tool to make respondents feel at ease to express their individual views, and the interviews were held in an environment where there were no external influences, interruptions, or third parties present. Second, before their participation in the study, the respondents were assured of the confidentiality of the interviews and the pseudonymization of the transcripts. Finally, all but one of the respondents were familiar with the interviewer as they had previous contact moments during the development of the dashboards. Growing familiarity with the interviewer has previously been associated with lessening the risk of socially desirable answers [36].

Our study did not allow for comparison between health care professionals who actively used the dashboards and those who did not, as we did not track the engagement with the dashboard (eg, opening or active use of the dashboard). As a result, we cannot fully distinguish whether limited impact of the dashboard reflects ineffectiveness or a lack of use. Future research incorporating engagement metrics could help elucidate this further.

Finally, a limitation of our study is that we were unable to disentangle the effects of the dashboard’s individual components on registration practices. All participating departments received a similar multifaceted dashboard incorporating multiple design features and data elements simultaneously, except for certain specifics, as illustrated in Table S2 in Multimedia Appendix 2. As such, we cannot ascertain which specific features, if any, were most influential in changing registration behavior. Future studies should consider evaluating dashboards with varying design elements and theoretical underpinnings to better understand which components are most effective in supporting structured data registration in CVRM.

### Conclusions

The extractability of CVRM indicators from structured fields of the EHR varied between departments and overall did not show improvement over time following the implementation of the dashboard in our LHS. This was explained by organizational, technical, and operational issues, including unclear responsibility for CVRM care, suboptimal technical knowledge of the EHR’s functionalities, limitations of the EHR system, and time constraints. These findings provide guidance on what aspects to consider for the extractability to be improved, which, in the end, will be beneficial for both clinical practice and scientific research using real-world data.

## Supplementary material

10.2196/71978 Multimedia Appendix 1 Location of the predefined structured fields in the electronic health record from which data were extracted for the dashboard.

10.2196/71978 Multimedia Appendix 2 Frequency of dashboards and content details of the dashboards per department.

10.2196/71978 Multimedia Appendix 3 Topic list and guide for the semistructured interviews.

10.2196/71978 Multimedia Appendix 4 Patient characteristics of the study population, by department.

## Abbreviations

CDSS: clinical decision support system
CVD: cardiovascular disease
CVRM: cardiovascular risk management
DBC: diagnosis billing code
EHR: electronic health record
HbA1c: glycated hemoglobin
HDL: high-density lipoprotein
LHS: learning health care system
RCT: randomized controlled trial
UCC: Utrecht cardiovascular cohort
UMC: university medical center
UPOD: Utrecht patient oriented database
