# Supplementary material for: Dashboards to Improve Extractability of Cardiovascular Indicators in a Learning Health Care System: Mixed Methods Study
Source: J Med Internet Res. 2025 Dec 16;27:e71978. doi: 10.2196/71978 (PMC12741949; doi:10.2196/71978)
Supplement: Multimedia Appendix 1 [file jmir-v27-e71978-s001.docx]

### Multimedia Appendix 1

Table S1. Location of the data fields in the EHR from which we extract the data for the UCC-CVRM dashboards.

| **Variable** | **Location in the EHR, in Dutch (and translated to English)** | **Screenshots of the EHR** | **Remarks** |
| --- | --- | --- | --- |
| Sex | Financieel/logistiek  > Patientgegevens  (Financial/logistics > patient characteristics) | **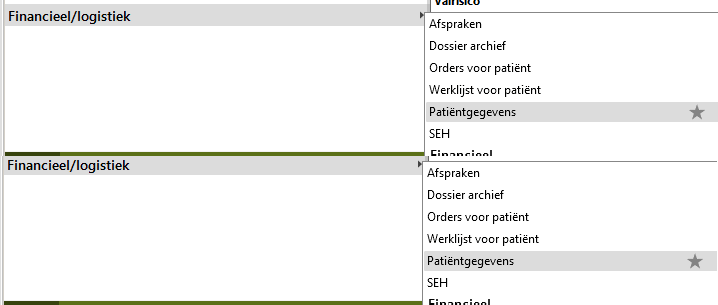** |  |
| Age | Financieel/logistiek  > Patientgegevens  (Financial/logistics > patient characteristics) | **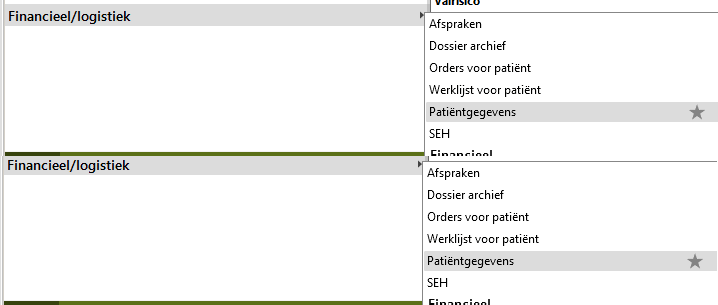** |  |
| Lab values (eg, cholesterol, HbA1c) | Uitslagen > Lab  Results > Lab | 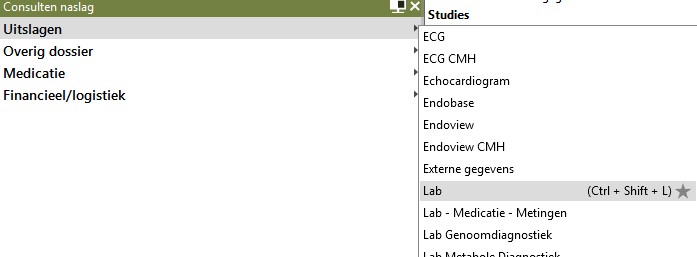 |  |
| Lab value –  eGFR | Not applicable | Not applicable | Calculated using the CKD- EPI formula based on age,  sex and creatinine |

| Measurements (BMI, blood pressure, etc.) | Uitslagen > Lab - Medicatie – Metingen  Results > Lab – Medication - Measurements | 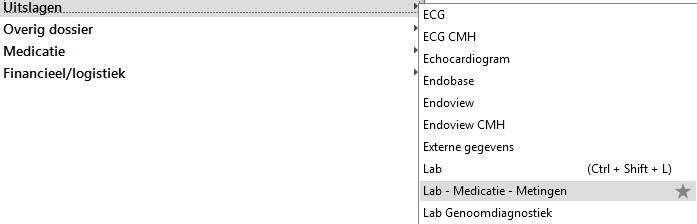 |  |
| --- | --- | --- | --- |
| Medical CVD history | Financieel/logistiek  > Verrichtingen  Financial/logistics > Procedures | 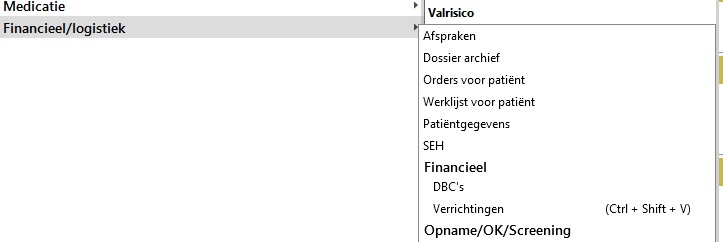 |  |
|  | Financieel/logistiek  > DBC's  Financial/logistics > DBC’s (Diagnosis Treatment Combination) | 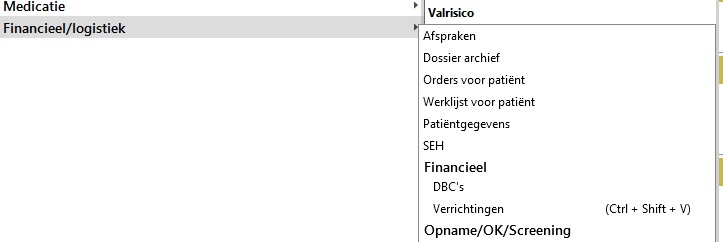 |  |

|  | Voorblad > Actieve diagnoses / Complicaties  Front page > Active diagnoses / complications | On the frontpage of the patient in HiX.  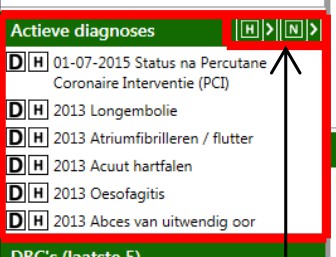 |  |
| --- | --- | --- | --- |
| Medication use | Medication | 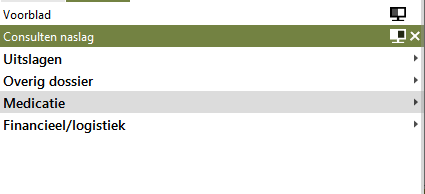 |  |

| Smoking | Anamnese (+) > Intoxicaties  Zorginformatiebouwsteen (ZIB)  Anamnesis >  Intoxications Health and Care Building Blocks, also called ‘Clinical Building Block’ | Anamnese (+). PEC needs to be selected as ‘Contact type’ for the intoxication building block to appear. |  |
| --- | --- | --- | --- |
