# Supplementary material for: Dashboards to Improve Extractability of Cardiovascular Indicators in a Learning Health Care System: Mixed Methods Study
Source: J Med Internet Res. 2025 Dec 16;27:e71978. doi: 10.2196/71978 (PMC12741949; doi:10.2196/71978)
Supplement: Multimedia Appendix 2 [file jmir-v27-e71978-s002.docx]

### Multimedia Appendix 2

Table S2. Frequency of dashboards and content details of the dashboards per department.

| **Department** | **Frequency** | **Content deviation from the standard dashboard** |
| --- | --- | --- |
| Cardiology | Monthly | Standard |
| Diabetology | Monthly | Standard + Medication use supplemented by use of GPL1-agonist and SGLT2-inhibitor use |
| Geriatrics | Quarterly | Standard + Age distribution added |
| Nephrology | Monthly | Standard |
| Neurology | Monthly | Standard |
| Vascular medicine | Monthly | Standard + Medication use supplemented by use of GPL1-agonist and SGLT2-inhibitor use + albumin creatinine ratio added as CVRM item |
| Vascular surgery | Monthly | Standard |
