# Supplementary material for: Dashboards to Improve Extractability of Cardiovascular Indicators in a Learning Health Care System: Mixed Methods Study
Source: J Med Internet Res. 2025 Dec 16;27:e71978. doi: 10.2196/71978 (PMC12741949; doi:10.2196/71978)
Supplement: Multimedia Appendix 3 [file jmir-v27-e71978-s003.docx]

### Multimedia Appendix 3

Table S3. Topic list and guide for the semi-structured interviews (translated from Dutch to English).

| **Question** | **Question behind the question (topics)** |
| --- | --- |
| 1. How long have you been working for the UMC Utrecht and in what position(s)? 2. Could you describe what an average day looks like for you?    1. How many patients do you see a day?    2. How much time do you have per patient?    3. How much time do you spend on administrative tasks such as registration? | Background: position & job description |
| 1. Are you familiar with the dashboard? 2. How was the dashboard used within your department?    1. Were the results shown in the dashboard used within your department?    2. Did you use the dashboard yourself? If yes, how? If no, why not? 3. Did your department set certain goals related to the information in the dashboards?    1. How was this implemented / acted upon? | Dashboard usage and goals |
| 1. How did you experience using the dashboard?    1. Which pros and cons did you experience? 2. How would you rate the dashboard? | Experiences with the dashboard |
| 1. Have the dashboards led to changes in your working method or in registering patient data? 2. What were these changes? | Influence on work practices |
| 1. Results from the quantitative data analysis show that structured registration of indicators did not improve nor worsened. Did you expect that?    1. Why did or did you not expect this? 2. What do you think could be the reasons for the changes (or lack thereof)? | Explanations for the quantitative results |
| 1. Do you think the dashboard could improve patient care?    1. How do you think it could improve patient care? [or] Why not? | Value of the dashboard |
| 1. According to you, what requirements does a dashboard need to meet to be relevant and of value for clinicians? | Value of the dashboard to the clinicians |

Table S4. Topic list (original Dutch version).

| **Vraag** | **Vraag achter de vraag** |
| --- | --- |
| 1. Hoelang werkt u al in het UMCU en in welke functie(s)? 2. Kunt u beschrijven hoe een gemiddelde dag eruit ziet?    1. Hoeveel patiënten ziet u op een dag?    2. Hoeveel tijd heeft u per patiënt?    3. Hoeveel tijd bent u kwijt aan de registratie van gegevens? | Achtergrond: Functie & invulling |
| 1. Bent u bekend met dashboard? 2. Hoe werd het dashboard binnen uw afdeling gebruikt?    1. Werden de resultaten uit het dashboard gebruikt binnen de afdeling?    2. Heeft u zelf het dashboard gebruikt? Zo ja hoe, zo nee, waarom niet? 3. Waren er binnen uw afdeling bepaalde doelen gesteld die aan de hand van het dashboard ‘getoetst’ konden worden?    1. Hoe werd hier invulling aan gegeven/ op gehandeld? | Dashboard gebruik en doelen |
| 1. Hoe heeft u het gebruik van het dashboard ervaren?    1. Welke voor- en nadelen heeft u ervaren? 2. Hoe beoordeelt u het dashboard? | Ervaringen met dashboard |
| 1. Hebben de dashboards geleid tot veranderingen in uw werkwijze / in het registreren van patiëntgegevens? 2. Welke veranderingen waren dit? | Invloed op werkwijze |
| 1. Sinds de dashboards zijn rondgestuurd zien we eigenlijk nauwelijks veranderingen in de registratie van patiëntgegevens. Deze zijn niet verbetert en niet verslechterd. Had u dat verwacht?    1. Waarom wel/niet? 2. Wat zouden volgens u de redenen hiervoor kunnen zijn?    1. Praktisch (te weinig tijd, etc.) /kennis/normatief/prioriteit? | Verklaringen voor kwantitatieve resultaten |
| 1. Denkt u dat het dashboard de zorg voor patiënten kan verbeteren?    1. Waarom wel / niet? | Waarde van het dashboard |
| 1. Waar moet een dashboard volgens u aan voldoen om relevant te zijn voor clinici? | Waarde voor de clinici |
