# Supplementary material for: Dashboards to Improve Extractability of Cardiovascular Indicators in a Learning Health Care System: Mixed Methods Study
Source: J Med Internet Res. 2025 Dec 16;27:e71978. doi: 10.2196/71978 (PMC12741949; doi:10.2196/71978)
Supplement: Multimedia Appendix 4 [file jmir-v27-e71978-s004.docx]

### Multimedia Appendix 4

|  | **Department A (n=489)** | **Department B (n=542)** | **Department C (n=1420)** | **Department D (n=555)** | **Department E (n=2416)** | **Department F (n=2846)** | **Department G (n=673)** |
| --- | --- | --- | --- | --- | --- | --- | --- |
| Age, med (IQR) | 76.0 (70.0-81.0) | 35.0 (30.0-53.0) | 55.0 (41.0-65.0) | 59.0 (42.0-71.0) | 65.0 (53.0-75.0) | 58.0 (40.0-70.0) | 67.0 (57.0-75.0) |
| Female, n (%) | 251 (51.3) | 417 (76.9) | 756 (53.2) | 260 (46.8) | 1182 (48.9) | 1331 (46.8) | 269 (40.0) |
| Smoker, n (%) | 33 (12.5) | 10 (6.2) | 64 (12.9) | 9 (18.8) | 251 (24.4) | 44 (12.6) | 34 (17.4) |
| BMI, mean (SD) | 26.6 (4.9) | 27.7 (6.2) | 26.8 (5.3) | 26.7 (5.5) | 26.0 (4.8) | 26.6 (5.0) | 25.5 (4.1) |
| SAP, mean (SD) | 149.2 (22.9) | 122.7 (18.5) | 143.4 (26.7) | 136.5 (23.1) | 153.1 (30.5) | 130.4 (22.6) | 141.9 (21.2) |
| LDL-c, med (IQR) | 2.4 (1.7-3.2) | 2.2 (1.7-2.8) | 2.7 (2.0-3.5) | 2.4 (1.8-3.0) | 2.5 (1.9-3.2) | 2.4 (1.8-3.2) | 2.3 (1.7-3.3) |
| Trig, med (IQR) | 1.5 (1.0-2.0) | 1.5 (1.0-2.5) | 1.3 (1.0-2.0) | 1.7 (1.2-2.4) | 1.3 (1.0-1.9) | 1.5 (1.1-2.2) | 1.8 (1.3-2.5) |
| HbA1c, med (IQR) | 40.0 (37.0-47.0) | 40.0 (34.0-60.0) | 37.0 (34.0-41.0) | 38.0 (34.0-44.0) | 39.0 (36.0-44.0) | 38.5 (35.0-45.0) | 37.0 (34.0-44.0) |
| Hb, mean (SD) | 8.3 (1.0) | 7.9 (1.1) | 8.7 (1.1) | 8.1 (1.2) | 8.6 (1.2) | 8.5 (1.3) | 8.2 (1.2) |
| Creatinine, mean (SD) | 87.1 (57.9) | 63.3 (41.2) | 78.5 (48.2) | 127.4 (69.3) | 81.4 (47.0) | 90.0 (57.7) | 198.6 (241.1) |
| eGFR(CKD-EPI), mean (SD) | 72.4 (19.9) | 110.4 (28.0) | 90.3 (21.9) | 61.3 (32.2) | 82.3 (23.8) | 82.1 (28.2) | 60.6 (34.1) |
| CVD history (yes), n (%) | 143 (99.3) | 50 (39.4) | 391 (100.0) | 123 (98.4) | 374 (99.5) | 505 (99.0) | 242 (99.6) |
| SCORE-NL, med (IQR) | 30.0 (22.0-37.0) | 2.0 (1.0-16.2) | 10.0 (2.0-28.0) | 18.0 (6.0-37.0) | 28.0 (10.0-37.0) | 9.0 (3.0-28.0) | 36.0 (25.0-40.0) |

Table S5. Patient characteristics of the study population, by department.

Notes: n = number of appointments; med = median; IQR = interquartile range; % = percentage; BMI = body mass index; SAP = systolic blood pressure; LDL-c = LDL-cholesterol; Trig = triglycerides; HbA1c = glycated hemoglobin; Hb = hemoglobin; eGFR (CKD-EPI) = estimated glomerular filtration rate using the Chronic Kidney Disease Epidemiology Collaboration equation.
